# Can Neuroscientists Test a New Physicalist Mind/Body View: DiCoToP (Diachronic Conjunctive Token Physicalism)?

**DOI:** 10.3389/fnhum.2021.786133

**Published:** 2021-12-17

**Authors:** Linda A. W. Brakel

**Affiliations:** ^1^Department of Philosophy, University of Michigan, Ann Arbor, MI, United States; ^2^Department of Psychiatry, University of Michigan, Ann Arbor, MI, United States; ^3^Michigan Psychoanalytic Institute, Farmington Hills, MI, United States

**Keywords:** mind/body problem, token physicalism, emergence, multiple realization, neuronal assembly, mental causation, Diachronic Conjunctive Token Physicalism

## Abstract

Given that disparate mind/body views have interfered with interdisciplinary research in psychoanalysis and neuroscience, the mind/body problem itself is explored here. Adding a philosophy of mind framework, problems for both dualists and physicalists are presented, along with essential concepts including: independent mental causation, emergence, and multiple realization. To address some of these issues in a new light, this article advances an original mind/body account—Diachronic Conjunctive Token Physicalism (DiCoToP). Next, puzzles DiCoTop reveals, psychoanalytic problems it solves, and some empirical evidence accrued for views consistent with DiCoToP are presented. In closing, this piece challenges/appeals for neuroscience research to gain evidence for (or against) the DiCoToP view.

## Introduction

Frontiers Research Topic, Psychodynamic Neuroscience, calls for a deepening dialogue between neuroscience and psychoanalysis. This is exciting, just as it stands. However, one further participant discipline, the philosophy of mind, could offer much toward addressing the conflicting mind/body views inhibiting progress both in psychoanalysis and neuroscience. It is within this added discipline that I attempt a contribution to this collection. The article consists of four sections, and then a final discussion, with conclusions and summary.

Section One explores essential problems embedded within the mind/body problem, arriving in Section Two at the view I devised (Brakel, 2013) and now advocate—Diachronic Conjunctive Token Physicalism (DiCoToP)^1^. Although the DiCoToP account resolves some of the issues associated with the mind/body problem, Section Three grapples with new puzzles that this particular mind/body account reveals and entails. Section Four, the final section, has two parts. In Part One, I aver that DiCoToP can go some distance in explaining two matters endemic to clinical psychoanalytic work but heretofore quite vexing. These are: (a) working through; and (b) the mechanism of therapeutic action after patients gain new knowledge through interpretation. Part Two, acknowledges that even if I am correct about these and more general aspects of DiCoToP’s explanatory power, much more than such coarse correlations are needed. Hence, after providing a brief account of some existing empirical studies whose findings can be taken to clearly support DiCoTop, the close of Part Two is essentially an appeal (and a challenge) to neuroscientists. Can neuroscientists apply cutting edge neuroscientific methods to explore in humans whether or not features of DiCoToP could obtain? Whatever the outcome, I would hope that this sort of empirical investigation would be a thoroughly interdisciplinary adventure, totally worth taking, as is the current research topic itself.

## Section One

### The Mind/Body Puzzle: Problems for Physicalists, and Dualists

‘‘Why physicalism?’’ is the first question every physicalist^2^ must consider—this, especially when dualism seems so appealing. Dualism’s appeal owes to its rather elegant solution to some basic and difficult mind/body problems for physicalists (especially physicalists of the reductive variety)—that of independent mental downward causation. Thus, as reductive physicalists maintain, (a) there is nothing over and above the physical (and the laws thereof), and (b) everything mental is at base physical; how then can mental goings-on—familiar processes of the mind, like qualia, beliefs, desires—be genuine causes of behavior, rather than just epiphenomena? Relatedly, dualism allows a clear, unproblematic grasp of such important concepts and phenomena as the Self, subjective first personal accounts, and even consciousness itself.

And yet, for physicalists, dualism presents its own insurmountable problem: If one embraces the most prevalent scientific view of a physical-only universe, the very nature of the mental—ontologically, metaphysically, as well as epistemologically—has not been, and perhaps cannot be, satisfactorily accounted for. Nonetheless, and notwithstanding this problem, there are two famous and compelling arguments for dualism. I will discuss them both in the section below, along with my claim that they do not work!

### The Dualists Have Two Great Arguments; But They Don’t Work

#### The Knowledge Argument

The Knowledge Argument was advanced by Frank Jackson in 1982. The main character is Mary, the color scientist. Jackson (1982, p.130) describes Mary as “…a brilliant scientist who is…forced to investigate the world from a black and white room via a black and white television monitor. She specializes in the neurophysiology of [color] vision and acquires…all the physical information there is to obtain about what goes on when we see ripe tomatoes, or the sky, and use terms like ‘red,’ ‘blue,’ and so on.” Jackson (p.130) then asks two pivotal questions: What will happen when Mary is released from her room and sees colors for the first time in her life? “Will she learn anything or not?” He concludes (p.130): “It seems just obvious that she will learn something about the world and our visual experience of it. But then it is inescapable that her previous knowledge was incomplete. But she had all the physical information. Ergo there is more to have than that, and Physicalism is false.”

This argument, although clever, compelling, and even convincing to many, is (on my view) fatally flawed. Indeed, one can assume Mary did not know what it’s like to experience color. But then comes the problematic part. For the argument to go through, one must also assume that knowing-what-it-is like type knowledge—so called acquaintance knowledge (or know-how knowledge)—is not part of physical knowledge. In other words, the argument assumes at the front end, exactly what it is trying to prove: namely that there exists knowledge that is non-physical.

#### The Zombie/Conceivability Argument

The Zombie/Conceivability Argument is presented concisely by Chalmers (2010, p.106). Here he paraphrases his earlier work (and that of others), setting up the Zombie Case as follows: “…It is conceivable that there be a system that is physically identical to a conscious being but lacks…that being’s conscious states. Such a system might be a zombie: a system that is physically identical to a conscious being but that lacks consciousness entirely.” He continues (p.107), that such a being/system

…will look identical to a normal conscious being from the third-person perspective. In particular…brain processes will be molecule-for-molecule identical with the original, and their behavior will be indistinguishable. But things will be different from the first-person point of view. What it is like to be…a…zombie will differ from what it is like to be the original being. [For] there is nothing it is like to be a zombie.

The next step in the Zombie Case, according to Chalmers (p. 107), is to acknowledge that since such zombie systems/beings are coherently imaginable with “no contradiction,” it can be inferred that they are a metaphysical possibility: “From here, it is inferred that consciousness must be non-physical. If there is a metaphysically possible universe that is physically identical to ours but that lacks consciousness, then consciousness must be a further, non-physical component of our universe.” Again, quoting Chalmers (p.107):

(1) It is conceivable that there are zombies.
(2) If it is conceivable that there are zombies, it is metaphysically possible that there are zombies.
(3) If it is metaphysically possible that there are zombies, then consciousness is non-physical.
(4) Consciousness is non-physical.

This argument for dualism too, is very appealing. And yet, there are serious problems embedded within its structure. Several authors, including Jackson (2003, pp.9, 30) and (Nagel, 1998, p.346), suggest that the Zombie Argument, turning on the conceptual conceivability of conscious-less human physical duplicates, is paradoxically a function of our own human concepts, and the limitations therewith (See also Hill, 1997). Thus, our powers of conceiving may outstrip both metaphysical reality and epistemological possibility. A physicalist can then mount a simple 6 step counter argument.

(1) Person-X and Zombie-X are molecule-for-molecule/neuron-for-neuron identical;
(2) Person-X’s consciousness is no more than a physical arrangement of these molecules/neurons;
(3) Zombie-X has the same arrangement of these same molecules/neurons;
(4) Hence, if Zombie-X does not have consciousness, this must be due to some consciousness-blocker.
(5) Any blocker must itself be physical, subject to physical laws (since there is nothing over-and-above the physical)^3^.
(6) The upshot: Either Zombie-X is not a zombie, and is conscious; or Zombie-X is not conscious, but not identical with Person-X.

With the hope that these refutations of two great dualist arguments have had some sway, let’s move on to some physicalist views.

### Non-reductive Physicalism: Appealing but Problematic

Non-reductive physicalism has promise. It is a physicalist/materialist view, and yet holds (as its name signals) that the mental is not reducible to the physical. It follows that non-reductive physicalism holds many of the advantages of dualism, while avoiding dualism’s biggest challenge—the ontologic nature of the mind and its mental goings-on. For non-reductive physicalists, the mind is strictly material/physical ontologically; but mental properties, e.g., qualia, the experience of sensations, the content of beliefs, consciousness itself, can neither be reduced to, nor explained by physical processes, not even physical laws. On the plus side, as with dualism, non-reductive physicalism allows such mental entities as Self, first person subjectivity, qualia, conscious attitudes and their contents (like beliefs and desires) to have real and independent downward causative powers.

However, there is a sizeable minus side too. If a physicalist maintains that there is nothing (no substance, or process, etc.) beyond or over-and-above the physical, what does the non-reductive solution really amount to? How is non-reductive physicalism different from property dualism? Next, and more important than the classificatory issue is the question of how these mental goings-on—irreducible-to-the physical—actually effect their novel, independent (non-epiphenomenal) causative powers? What laws of nature apply?^4^

Emergentism might help the non-reductive physicalist. In simplest terms, emergentism holds that “macroproperties” arising from a number of “microproperties” conjoined, can have autonomous causal effects. For example, many H_2_O molecules combined constitute a macroproperty, emerging from a combination of many single H_2_O molecules—the microproperties. Note that the macroproperty, water, has properties, different and novel, from the underlying microproperties—the single H_2_O molecules. Here the macroproperty, water, is a fluid, a liquid, it is wet. These are emergent properties, and water is causally autonomous and independent from those of the underlying single H_2_O molecule microproperties.

Applying this to the mind/body puzzle—emergentists aver that mental macroproperties emerge from physical microproperties. Searle (1992, p.112) states:

…consciousness is a causally emergent property of systems. It is an emergent feature of certain systems in the same way that solidity and liquidity are emergent features of systems of molecules. The existence of consciousness can be explained by the causal interactions between elements of the brain at the micro level, but consciousness cannot itself be deduced or calculated from the sheer physical structure of the neurons without some additional account of the causal relations between them.

This kind of sanctioned emergence Searle calls “emergent1” and he (p.112) makes a sharp distinction between such allowable emergent1 phenomena and another sort of emergence which Searle abjures. Here is Searle (p.112) on “… a much more adventurous conception, call it ‘emergent2.’ A feature F is emergent2 iff [if and only if] F is emergent1 and has causal powers that cannot be explained by the causal interactions a, b, c…’’ Searle continues (p.112), ‘‘If consciousness were emergent2, then consciousness could cause things that could not be explained by the causal behavior of the neurons.’’ This would be an impossible outcome that Searle rejects^5^.

But Searle’s important discussion of emergentism has not actually solved the problem. Yes, Searle acknowledges that the existence of consciousness is reductively accounted for physically, but to repeat the last clause of his last quoted sentence just above: “…but consciousness cannot itself be deduced [reduced to] or calculated from the sheer physical structure of the neurons without some additional account of the relations between them.” This is true, but Searle does not answer two questions that immediately follow: What are these accounts? And more broadly, can consciousness indeed be deduced or calculated from additional accounts of the relation of neurons, even in principle? If yes, then reductive, rather than non-reductive, physicalism is rescued; but is its cost too high with respect to autonomous mental causal powers? On the other hand, if consciousness cannot be so calculated, the notion of independent mental causation seems vindicated; but do these autonomous mental powers arise in the forbidden emergent2 fashion?

In the next section, I will present my own view, which features mental goings-on as emergent1 phenomena, along with the sort of independent causal powers seen with other emergent1 macroproperties, such as the familiar example of water’s liquidity and H_2_O molecules?

## Section Two

### Diachronic Conjunctive Token Physicalism: DiCoToP

#### Why Token Physicalism, Not Type Physicalism?

In continuing to explore reductive physicalism, there is first another terminological issue: Reductive physicalism/materialism can also be called identity theory. Next, there is a substantive categorization matter: There are two sorts of reductive physicalists—type and token physicalists. J.C. Smart (1959, p.156), advocates for reductive physicalist “brain process theory [both type and token]” on the basis of “the principles of simplicity and parsimony.” Hill (1991, p.6), a type materialist himself, holds that both type and token physicalists “…maintain that we obtain a simpler and more straightforward picture of the universe if we assume that sensory [mental] events are identical with physical events.” Elaborating this further, Hill (p.22) discusses reductive physicalists as having the scientific advantage of “the best explanation principle” whereby, “If a theory provides a good explanation of a set of facts, and the explanation is [as good as or] better than any provided by a competing theory, there is good and sufficient reason for believing that the theory is true.”

Now as to type vs. token physicalism, Hill (p.11) explains: “*Type materialism* is like token materialism in claiming that sensory [mental] events are identical with physical events. However, [different from token materialism], it also claims that there is a set of physical characteristics with which qualitative [mental] characteristics are universally and lawfully correlated…” Continuing, Hill (p.35) goes on to assert that “…type materialism is simpler than dualism because it postulates fewer *events*…[and] type materialism is simpler than double aspect theory because it postulates fewer *facts* [Hill’s italics throughout].” And as far as type materialism over token becoming the more popular view, it is understandable because type materialism brought a universal, lawful regularity to the vexing mind/body problem: predictable mappings of mental state types of a certain character onto brain states types of a certain character. What could go wrong? The answer is multiple realization, the topic of the next subsection.

#### Multiple Realization

First articulated in a seminal article by Putnam (1967/1975), the Multiple Realization Argument (MR), and its consequences for type physicalism is described in some detail by Kim (1993). According to Kim (p.179) The MR argument advances the idea that given that “…any mental state…can be ‘physically realized’ in many diverse types of organisms and physical structures (e.g., humans, mollusks, crustaceans, and perhaps Martians and robots) so that, as a matter of empirical fact, it is extremely unlikely that some uniform physical state exists to serve as…physical correlate [to that mental state].” What follows then, according to Kim (p.272):

… [Since] any psychological event-type can be “physically realized” or “instantiated” or “implemented” in endlessly diverse ways, depending on the physical-biological nature of the organism or system…it [is] highly implausible to expect the event[-type] to correlate uniformly with, and thus be identical with [or reducible to] some “single” type of neural or physical state.

This conclusion from the MR argument made a deep impact upon those working on the mind/body question. Kim (1993) characterizes its effect as profound and devastating to type materialism (p.309): “‘[T]ype materialism’ is standardly thought to have been definitively dispatched by MR to the heap of obsolete theories of mind.” Indeed, because of this argument, type materialists seem to have retrenched. Certainly they limited their focus, as talk of type materialism narrowed to type materialism within a species. Thus there would be human type pain (Mental-_H–P_) and human type brain circuits (Physical-_H–BC_); these not necessarily similar to mollusk type pain (Mental-_Mol–P_) and mollusk nervous tissue (Physical-_Mol_-_NT_); and Martian type pain (Mental-_Mar–P_) and Martian type physical type pain realizer (Physical-_Mar–PR_); all of these also not necessarily similar to one another.

But, as I remarked in 2013 (Brakel, pp.75–76) and paraphrase here: MR is problematic even for species-specific type physicalists. Neuroscientists have long established that among different individual humans there are various brain circuits involved in the same mental property—sometimes even gross differences in brain areas. Along with these micro and even macro brain differences across individuals, all realizing the same mental property/event, there are variations within a single individual—this even more problematic for type physicalists. According to Bechtel and Mundale (1999, p.176) within any one person “… the same psychological state can be realized by different brain states…a many-to-one mapping from brain states to psychological states.” The term for this is “biological degeneracy,” which Edelman and Gally (2001, p.13763) define as “…the ability of elements that are structurally different to perform the same function or yield the same [contentful] output.” Noppeney et al. (2004, pp.440–441) report that sometimes such within-individual degenerate plastic brain changes can be dramatic in preserving important psychological functions. Getting even more fine-grained and specific, Figdor (2010) explains (p.435): “On the neuroanatomical side, single cells, neuronal populations, anatomical areas, or anatomical networks are among the ‘structural elements’ that may appear in degenerate mappings.” (See also Price and Friston, 2002; Friston and Price, 2003; Noppeney et al., 2004).

Undoubtedly the brain’s degeneracy and its plasticity are of great import for survival and for evolutionary success. Yet, these current scientific developments severely compromise any type physicalist view of the mind/body question. It becomes increasingly apparent that even the more limited local species-specific type physicalist cannot rely on type-type identity or type-type reduction even within the brain of a single individual, much less across the brains of an entire species. No one-to-one physical type to mental type correlation can be found to exist.

The usual reaction among philosophers to this daunting challenge to type materialism has been to turn back to a non-reductive version of physicalism, neglecting token materialism, which in my view deserves much further investigation. Indeed, in the next subsection another issue will be presented, which while seemingly problematic, also admits of possible solutions offering potential advantages for token physicalism.

#### The Question of Scale

An understanding of multiple realization leads to another potential problem for physicalist-identity mind/body solutions, token as well as type. That is the issue of scale. There is an obvious mismatch between a mental process/event—e.g., a particular belief—and the myriad of neurons that underlie such a belief, even in a single person at a single time. The brain processes are much more fine-grained. Now add to this the fact that the same specific belief can be realized on a number of different occasions, each with its own neuronal assembly. Some such assemblies are found within degenerate mappings, as described above (See also Friston, 1997; Price and Friston, 1997). But even when neuronal assemblies occur within a localized neural circuit, there is still a one-to-many mismatch. Neuroscientists Papadimitriou et al. (2020, p.14464) characterize neuronal assemblies in general as “…large populations of neurons believed to imprint [particular] memories, concepts, words, and other cognitive information.” They further describe these as “randomly connected,” demonstrating plasticity.

Given this mismatch, how can token physicalism—which essentially holds that one particular mental event is identical with a physical event—hold up? Stephen Yablo (1992, p.256, 271) proposes a view of the relation between mental and physical properties and events, which can be helpfully applied to token physicalism. Yablo (1992, p.256) briefly outlines his account of determinable/determinate relations as follows: “Necessarily, something has a mental property iff [if and only if] it has also a physical determination of that property.” This, he explains, “…is an instance of the standard equation for determinable and determinates, generally, namely, that something has a determinable property if it has some determinate falling thereunder.” The color blue for example is a determinable with various shades of blue—navy blue, oxford blue, pale blue, gray blue, sky blue, turquoise blue, midnight blue, royal blue—being among its determinates. Applying this to the question at hand, a particular belief (and its content) would be the determinable, while several different neuronal assemblies would be its various determinates.

#### Composites and Conjunctions

The idea of a single determinable with multiple determinates raises another interesting matter. Is a determinable a composite of all of its determinates? Is it a conjunction? Is it different for various determinable/determinate combinations? The question is hard to answer for a determinable like blue—but blue seems to be more of a mixture of the different blues; a composite, rather than a conjunction. The question is more easily settled for another sort of determinable, The New York Yankees. Since its inception as the team called ‘‘The New York Yankees’’ in 1913,^6^ until its current instantiation in 2021, the New York Yankees are a conjunction (not a composite) of all of the New York Yankee teams during those years. On the other hand, all of the players playing for a specific Yankee team, e.g., 1960, comprise a composite—the 1960 New York Yankees. But, interestingly if one, two, or even all the players had been traded, the 1960 New York Yankees would still have been the 1960 New York Yankees, still a composite, but a different one, consisting of different parts, and different tokens.

Of course, the question here is how do these matters apply to the mind/body problem and token physicalism. I will attempt to answer this in the next subsection.

### Diachronic Conjunctive Token Physicalism (DiCoToP)

I have posited (Brakel, 2013, Chapter 3) a novel token physicalist view of the mind/body problem—Diachronic Conjunctive Token Physicalism (DiCoToP). It features a brain-based view of mental content in which each singular mental event (i.e., a particular belief with a specific mental content^7^) occurring at time-t, exists as a brain event at time-t, consisting of an assembly of neurons, along with whatever neurochemical processes facilitate their connection. This is a reductive token physicalist view, which as such means that at time-t, synchronically, there is nothing over and above these brain goings-on as far as the specific mental event is concerned. Further, each additional singular instance of the same mental content (each token)—take for example, “My dog is fun”—is populated by a different network of neurons, perhaps even a different neuronal network, because it occurs at a different time, in a different place, and within a different context. Sometimes the neural assemblies at times-t and t + 1 or t + 20 vary only slightly; sometimes they are quite different. In any case, the sum of all of these instances of this content, i.e., all of their variable neuronal assemblies—neurons, networks, circuits—the conjunction, over time, diachronically, constitutes the mental content.

On this account, the mental event/process would be the determinable and its various neuronal ensemble instantiations would be the determinates. Following Yablo (1992, p.259), this understanding admits of at least a partial resolution of the mental causation problem: “…determinates and their determinable…are not causal rivals…” And Yablo adds (p.272) “…rather than competing for causal honors, determinable and their determinates seem likelier to share in one another’s successes.”

But even if these determinables—the mental goings-on—do have autonomous causal powers, there remains the still vexing question of the relation of the physical to the mental, namely how is it that mental goings-on, like consciousness, arise from its physical-only base?^8^

Re-exploring emergence (particularly the sanctioned emergent1 process) now with the DiCoToP model in hand, a solution (at least a partial one) to this overarching puzzle might be achievable. But, what is more certain, is that emergent1 processes viewed in terms of the DiCoToP mind/body model, can add substantial evidence for autonomous mental causation. Recall that macroproperties consisting of collections of microproperties do have real and novel causal powers. A big collection of H_2_O molecules has new properties—it is a fluid, it is wet—and with these properties has causal powers independent from single H_2_O molecules. Diachronic Conjunctive Token Physicalism maintains that a specific singular mental content repeated over time—e.g., the belief “my dog is fun”—should be considered a macroproperty consisting of the conjunction of the many physical neuronal assembly microproperties entrained over the numerous instances of this particular mental content. Indeed, this fits with Searle’s (1992) suggestion, described above, that Ms are emergent1 macroproperties, micro-based upon Ps; and their relation can therefore, in principle, be explained in the usual way by normal science.

But, to be fair, this account of Ms and Ps is not a universally endorsed. Kim (1998, p.98), for one, does not consider an M to be a macroproperty micro-based on Ps; and even Searle (1992) contests the emergent1 solution as explanatory for first person ontology. That acknowledged, let’s consider what is different in the relation of Ps to Ms, on the one hand, and H_2_O molecules to the wet fluid we know as water, on the other. The difference, I aver, is that physical chemists know how the multiple molecules of H_2_O, when accumulated give rise to wet water. They can understand the organization of the many molecules within a structure, the Brownian movements of the molecules under temperature and pressure conditions, etc., On the contrary, even if DiCoToP proves to be correct, we would still have no good understanding of the “how” relation between mental contents as a macroproperty and the myriad neuronal assemblies that are its microproperties. Hence, the final subsection of this work will essentially be a mixture of plea and challenge to neuroscience colleagues to work on the “how” within the DiCoToP account. But first, in the section just to follow are some new puzzles the DiCoToP view brings to light.

## Section Three

### New Problems Revealed/Entailed by Diachronic Conjunctive Token Physicalism

With Diachronic Conjunctive Token Physicalism in place, new puzzles come to the fore. To demonstrate, let’s start with a thought experiment. Suppose that water’s liquidity/wetness which seemingly owes to a very large collection of H_2_O molecules “really” owes to the perceptual capacity of the subject experiencing the wetness. In other words, suppose that for a very different sort of experiencer (one that is microscopically small) only two or three H_2_O molecules would suffice as a sufficient microproperty base for the emergent1 macroproperty, water, to arise—this, with its liquidity, wetness, and most importantly, its independent causal powers. The parallel question immediately presents itself: Would a small number of neural assemblies, even just two, allow this imagined tiny experiencing subject to experience consciousness, and allow mental independent (not epiphenomenal) causal efficacy?

Related to this is the Sorites Paradox, a paradox demonstrating the inherent vagueness (lack of specification of the extension) of certain concepts. Here are two simple examples involving the concepts “baldness” and “the heap.” If someone is bald and gets 1 more hair, is he still bald? What about 2? What about increasing by 1 hair iteratively? Likewise, does 1 grain of sand constitute a heap? No, what about 2? What about increasing 1 grain-at-a-time for n-times? Which specific hair makes the difference between baldness and no longer bald? Which grain is the one that constitutes the heap? No one of them can be that potent, and yet at some point there is a heap^9^. Now let’s apply this first to the uncontested macroproperty, water. How many H_2_O molecules are necessary for us to perceive H_2_O as wet, liquid water? Next, how many neurons and neuronal assemblies are necessary for consciousness to be recognized as such? In addition to the ‘‘recognized-as-such-by-whom’’ problem outlined just above, we now have introduced the notion that the very concept of consciousness might be vague, unspecifiable^10^,^11^.

Both of these problems might lead one to the panpsychism family of materialist mind/body views, in which (a) physicalism holds; (b) conscious experience is the only thing we really know for sure; and (c) even the most basic physical particles (including their subatomic constituents) can be mental/conscious (Again, see especially Strawson, 1994, 2009). But as I’ve outlined earlier (footnote 4), these views also have major problems. Here are two: (1) Either there must be an accounting for whatever aspects are non-conscious/non-experiential, if any such parts are posited; or (2) If there is nothing that is non-conscious, the panpsychist needs to explain why it is that our human conscious experience is not experienced until these fundamental “conscious” particles undergo much combinatory work. Further, and of no less importance, the nature of these combinatory processes ought to be outlined.

Paradoxically, it is these somewhat esoteric philosophical puzzles revealed by DiCoTop that can return us to something potentially more solvable—although still very difficult. Here then is the central question:

Given (a) that many H_2_O molecules are needed for the emergent1 property of liquidity and the subjective experience thereof; and (b) that the “how” of liquidity/fluidity can be understood in terms of the principles of physics and physical chemistry.

Q: Can a similar understanding about how the myriad neural assemblies align/combine in emergent 1 fashion to yield consciousness?

So far no, but eventually, with more advances in neuroscience, couldn’t the answer evolve: first to “not yet” and later to “yes!”? Meanwhile, the Diachronic Conjunctive Token Physicalist view does now allow some explanation for some heretofore perplexing aspects of psychoanalysis. It is to these I next turn.

## Section Four

### Part One: DiCoToP Can Explain A Problem Endemic to Clinical Psychoanalyses

“Working-through” is a concept familiar to psychoanalysts. It represents an important process present in almost every psychoanalytic treatment. Working-through is especially significant theoretically as it can be seen as a shorthand version of perhaps one of the least understood problems for clinical psychoanalyses—namely, what is the mechanism of therapeutic action after interpretative work facilitates new knowledge? To the extent this is the case, comprehending working-through will aid in understanding this second more extensive problem. But even the “simpler” mechanism of working-through, specifically how it actually takes place, has not been well grasped. Thought to be related to grieving, the basic notion is that reviewing/re-working painful, problematic or neurotic contents from different angles helps to effect actual change. The mind/body account I’ve advanced here and originally in Brakel (2013), Diachronic Conjunctive Token Physicalism, in its brain-based view of mental content, could go some distance in explaining what the working-through mechanism might actually entail and therefore how working-through might actually work.

To review what I’ve said above (Section Two, infra pp.5-6), on the DiCoToP account, every singular mental event (including a specific mental content, e.g., a particular belief) exists synchronically (at a time) as a brain event consisting of an assembly of neurons, along with whatever neurochemical processes facilitate their connection. Further, each singular instance of the same event/same mental content (each token) is constituted by a slightly different population of neurons, a slightly different neuronal assembly,^12^ insofar as it occurs at a different time and likely a different place. The sum of all of these instances of this content, i.e., all of their slightly variable neuronal networks (the conjunction), over time (diachronic), comprises the mental content.

Now, let’s take a neurotic belief with a clear and simple mental content “All spiders are dangerous and should be avoided.” How would the working-through process actually occur according to the DiCoToP model of mental events? It seems clear that dealing with the phobic pathology that this specific mental content represents would require much re-aligning over a great number of neuronal assemblies, networks, and circuits, all summed over time. In other words, considerable experiential re-workings of the particular mental content would be needed—this, in most of the myriad contexts in which the neurotic belief appeared.

Working-through more complex neurotic symptoms, composed as they are of diverse and variegated neurotic beliefs, would clearly entail more realignments, more re-workings, more time, much of it taking place after gaining new knowledge from even the most successful interpretations. Diachronic Conjunctive Token Physicalism is then a mind/body view that can help explain not only working-through and the post-interpretative mechanism of therapeutic action, but also why analyses take so long, and why post analytic self-analysis is rarely optional.

### Part Two: Can Neuroscientists Provide Evidence for (or Against) DiCoToP?

There is actual neuroscientific evidence for the sort of neuronal assemblies underlying mental processes that the Diachronic Conjunctive Token Physicalism (DiCoToP) model proposes (See Carillo-Reid and Yuste, 2020 for an overview). The mental processes include learning following sensory stimulus (Litwin-Kumar and Doiron, 2014); visual perception (Miller et al., 2017); tactile discrimination (Deolindo et al., 2017); appetitive learning (Brebner et al., 2020); formation of memories, concepts, words (Papadimitriou et al., 2020); pattern completion (Carillo-Reid et al., 2021); and learned reaction-time tasks (Narayanan et al., 2005). These studies all have shown (a) central stability in neuronal ensembles; and (b) ensembles that nonetheless include various levels of plasticity. These studies document that shifting neuronal membership occurs often, further demonstrating the type of redundancy seen in many biological systems (See Edelman and Gally, 2001, p.13766; Narayanan et al., 2005; Hiratani and Fukai, 2018). Moreover, these research findings have included degenerate neuronal arrays at the level of disparate circuits, reflecting an even greater degree of plasticity.

Note that in the experiments cited, the methods employed to study the neuronal assemblies—including their original formation, their variability, and resultant stability amidst the changes—have involved sophisticated two-photon calcium imaging/optogenetics and complex statistical modeling. With these tools, researchers documented important aspects of neuronal assemblies, including variability sometimes seen even in their synaptic connections,^13^ as well the more familiar specific neuronal membership shifts.

However, all of the optogenetic imaging and their encouraging results, were of necessity gathered on non-human animal subjects. What are the implications for human consciousness? The statistical modeling studies, on the other hand, were directly applied to human mental goings-on, and they are certainly informative. Returning to the animal studies, the subjects were clearly conscious in their responses to perceptual stimuli and various learning tasks. This means that the fine-grained (neuronal assembly, neuron, and even synapse-level) imaging results do reflect mental activity, which should be generalizable to human animals. And yet…

Here is one possibility offered by this author (a not-even-neophyte neuroscientist; but a psychoanalyst/philosopher) in order to test DiCoToP more specifically. Could the research methods described above (optogenetic imaging) be employed to study particular mental contents more directly in appropriate non-human animals? For example, two simple belief-like attitudes for a mouse might be selected: (1) “that is frightening;” and (2) “that is appealing.” Then, many versions of each of those content-rich beliefs would be delivered to each mouse-subject (e.g., 10 different mouse-subjects) in many iterations (20) during a single session, and then in succeeding sessions over some duration of time (e.g., 2 weeks)? Would imaging results compared within subjects and between subjects—both with regard to trials at-a-time, and those over-time—prove revealing?

And now, for the real point of this section, really of this whole article: I wrote it in the hopes of providing a challenge and registering a plea to neuroscience colleagues to devise human experiments offering empirical brain-based (perhaps neuronal assembly and neuron level) evidence for or against DiCoToP (as well as other competing mind/body views).

## Discussion: Summary and Conclusion

Any investigation of the perpetually vexing mind/body problem should not exclude a philosophical exploration, especially in light of the claim that neuroscientists and psychoanalysts hold clashing positions inhibiting their interdisciplinary collaboration. Thus, this article adds the philosophy of mind to the endeavor set forth as the “Frontiers Research Topic: Psychodynamic Neuroscience.” This piece begins with a series of questions: Why should one be a physicalist, when dualism can seem so right? Why should one be a reductive physicalist, when non-reductive physicalism has the appeal of dualism, without some of its major problems? And finally, why token physicalism, when type physicalism has been much more popular?

Asking and answering these questions provides a review of some of the most important aspects of the mind/body dilemma. Among the issues taken up was the matter of independent causation. If the mind and the mental are properly reduced to the physical, how can mental goings-on have real autonomous, downward causal power? Are they not just epiphenomena? Emergence as a possible solution was discussed, along with the problems it does not resolve. Next, multiple realization, a concept that is truly pivotal for neuroscience and mind/body physicalists, was explored, and found to be particularly relevant as it has become a central feature in the mind/body view I developed in 2013, and outlined in the current article. Termed Diachronic Conjunctive Token Physicalism (DiCoToP), this account can resolve many of the outstanding mind/body problems. True, the DiCoTop account also brought to the fore a few new vexing philosophical puzzles. But after wrestling with these, the article took a more practical turn.

Several empirical studies, of two sorts—statistic modeling of neurological phenomena at the level of the neuron and neuronal assembly; and imaging studies on non-human animal subjects on fine-grained brain processes—were seen to provide evidence for essential aspects advanced by the DiCoToP account. This article ends with a challenge that is at the same time an appeal: Can neuroscientists devise empirical experiments with human subjects in order to gain evidence for (or against) the Diachronic Conjunctive Token Physicalist mind/body account?

## Data Availability Statement

The original contributions presented in the study are included in the article/supplementary material, further inquiries can be directed to the corresponding author/s.

## Author Contributions

The author confirms being the sole contributor of this work and has approved it for publication.

## Conflict of Interest

The author declares that the research was conducted in the absence of any commercial or financial relationships that could be construed as a potential conflict of interest.

## Publisher’s Note

All claims expressed in this article are solely those of the authors and do not necessarily represent those of their affiliated organizations, or those of the publisher, the editors and the reviewers. Any product that may be evaluated in this article, or claim that may be made by its manufacturer, is not guaranteed or endorsed by the publisher.

## Footnotes

1 Note, I have taken up the mind/body issue in much greater length and depth in Brakel (2013). Much will be abbreviated and condensed here.

2 Physicalism is synonymous with Materialism in the mind/body context; as such they will be used interchangeably here.

3 Blockers, described by Leuenberger (2008), are necessary if one is actually conceiving of initial physical identity between Person-X and Zombie-X; this, whether or not one is aware of conceiving of blockers! But Chalmers (2010, p.164) tries to block blockers, “[W]hen we conceive of the zombie world…we conceive of a world with physics and *nothing else* [Chalmer’s italics]; no blockers.” Hence Chalmers not only argues that consciousness is non-physical, he extends this to blockers. We must then ask, why would blockers be outside the world of physics?

4 Similar problems, and more, arise for dual-aspect monists, particularly those who suggest that the monism endorsed is not strictly physical, but algorithmic or symbolic. Also, there are panpsychists, who also hold that there is nothing beyond the physical, but turn things upside down positing that it is the experiential/mental that is clearly and incontrovertibly physical. Further, that this is the case fundamentally, perhaps right down to the level of atomic (and subatomic) particles (See especially Strawson, 1994, 2009). Difficulties here arise in (a) accounting for whatever is non-conscious/non-experiential, and (b) explaining the seeming lack of conscious experience until the fundamental particles undergo much combinatory work. But an examination of these issues is beyond the scope of this article.

5 Continuing with Searle (p.112) on the very impossibility of emergent2 phenomena, he states: “…it seems unlikely that we will be able to find any features that are emergent2, because the existence of any such features would seem to violate even the weakest principle of the transitivity of causation.” Strawson (2006, pp. 69–71) also dismisses emergent2 emergence, labeling it “radical emergence.”

6 This team played under different names from 1901–1903 and 1903–1913.

7 I chose mental content for ease of explanation and potential ease of research investigation. A reviewer made the incontrovertible point that emotions have much to do with shaping cognition, and, that actions are integrally involved with the workings of the brain. Since I totally agree, additional (or other) empirical research would be welcome. For example, perhaps neuronal assemblies responding to basic positive vs. negative stimuli (pain vs. pleasure) would admit of neuroscientific investigation with equal (or perhaps greater) facility than the neutral content I put forward. Actions too—both spontaneous and directed—could be good sources for neuroscientific studies of plastic neuronal assemblies.

8 Yablo (1992, p.256n) also acknowledges this unsolved problem

9 For more on vagueness and the Sorites Paradox, including basic references, see Brakel (2010), Chapter 3, pp.53–87.

10 So, is the problem of consciousness only a problem for creatures like us, with problematic vague concepts, and the constraints (size and otherwise) of our particular perceptual capacities? In other words, is this a mind-dependent subjective problem writ large? But note, we are creatures like us—we are us—and of course we want to solve the problem for us!

11 On the other hand, as suggested by a reviewer, consciousness may be a threshold phenomenon, and as such not so susceptible to the sorites problem.

12 Sometimes the neuronal collections can show greater ensemble variation; indeed, even diverse neural circuits can at times be entrained.

13 For synaptic redundancy, see Hiratani and Fukai (2018).
